# Supplementary material for: Temporally resolved analyses of aperiodic features track neural dynamics during sleep
Source: Commun Psychol. 2025 Nov 19;3:160. doi: 10.1038/s44271-025-00334-2 (PMC12630853; doi:10.1038/s44271-025-00334-2)
Supplement: Supplementary file 2 — Supplementray Information [file 44271_2025_334_MOESM2_ESM.docx]

# 10. Supplements

## 10.1. Supplemental Tables

| **Article** | **Frequency band** |
| --- | --- |
| Alnes et al., 2023 [69] | 2 - 20 Hz |
| Ameen et al., 2023 [70] | 1 - 45 Hz |
| Andrillon et al., 2020 [71] | 0.5 - 20 Hz |
| Bódizs et al., 2021 [39] | 2 - 48 Hz |
| Demirel et al., 2021 [29] | 30 - 45 Hz |
| Favaro et al., 2023 [72] | 1 - 20 Hz |
| Feinberg et al., 1984 [8] | 0.5 - 12 Hz |
| G. Horváth et al., 2022 [11] | 2 - 48 Hz |
| Kozhemiako et al., 2022 [13] | 30 - 45 Hz |
| Lendner et al., 2020 [14] | 30 - 45 Hz |
| Lendner et al., 2023 [15] | 25 - 45 Hz |
| Maschke et al., 2023 [73] | 1 - 45 Hz |
| Miskovic et al., 2019 [16] | 0.5 - 35 Hz |
| Pereda et al., 1998 [8] | 3 - 30 Hz |
| Schneider et al., 2022 [18] | 2 - 48 Hz |
| Wen and Liu, 2016 [74] | 1 - 30 Hz |

***Supplementary Table 1. The results of the literature search.*** *A list of publications that investigated the spectral exponent in the human brain during sleep. These articles had a clear mention of the frequency range used to fit the model. Other studies that did not explicitly mention the range were not included. One study [73], incorporated the use of a knee model in its analysis. This study reported an enhanced model fit when utilizing the knee model compared to the fixed model.*

## 10.2. Supplemental Figures:


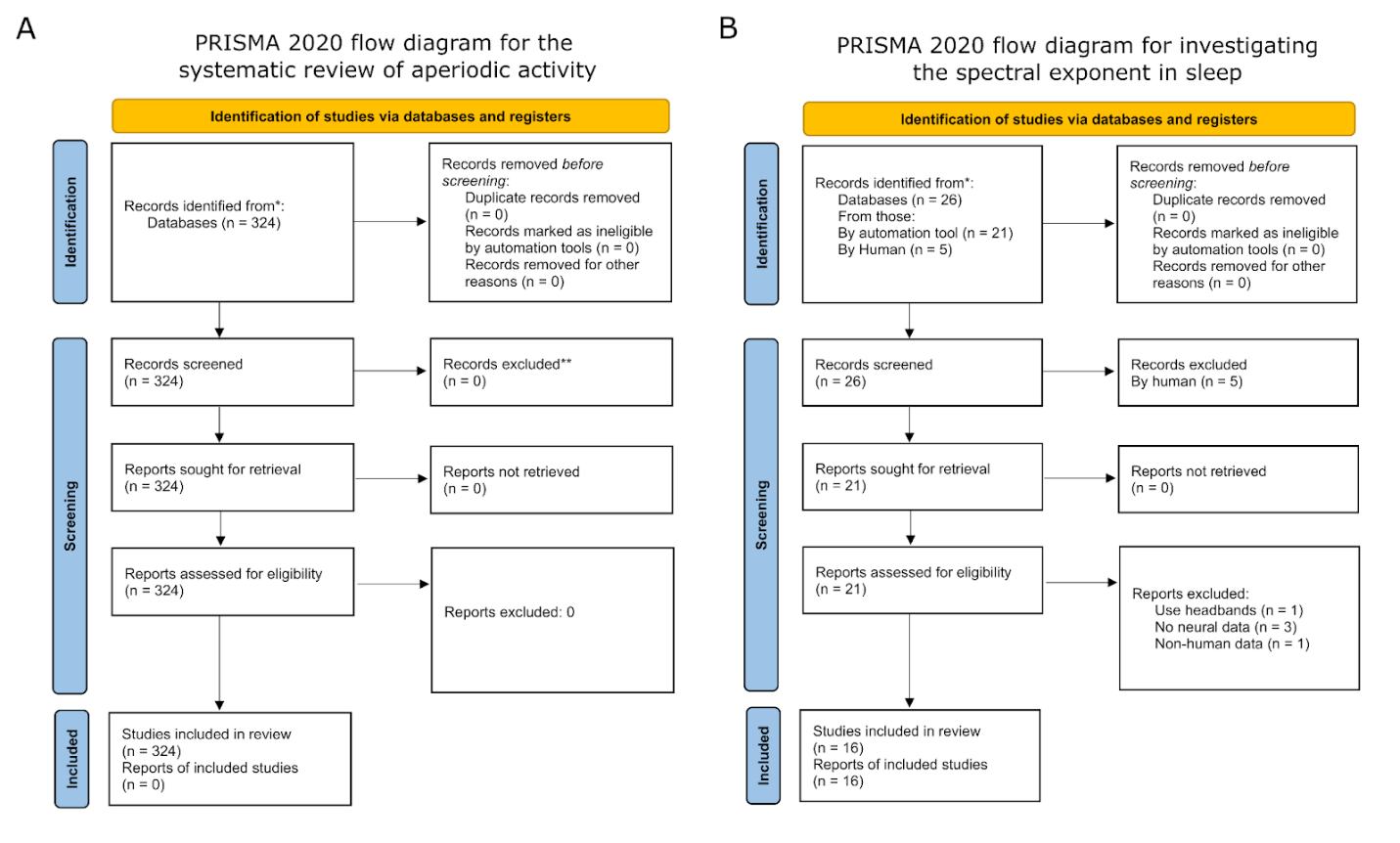
***Supplementary Figure 1. PRISMA 2020 Flow Diagrams for Systematic Reviews****. (A) PRISMA flow diagram summarizing the process for the systematic review of aperiodic activity in brain and sleep studies. A total of 324 records were identified from databases. No records were excluded, removed, or deemed ineligible, resulting in all studies being included in the final review. B) PRISMA flow diagram for investigating the spectral exponent in sleep. A total of 26 records were identified, with 21 identified through automated tools and 5 manually. Five records were excluded during screening, resulting in 21 reports being assessed for eligibility. Of these, 5 were excluded for various reasons, leaving 16 studies included in the final review.*

***
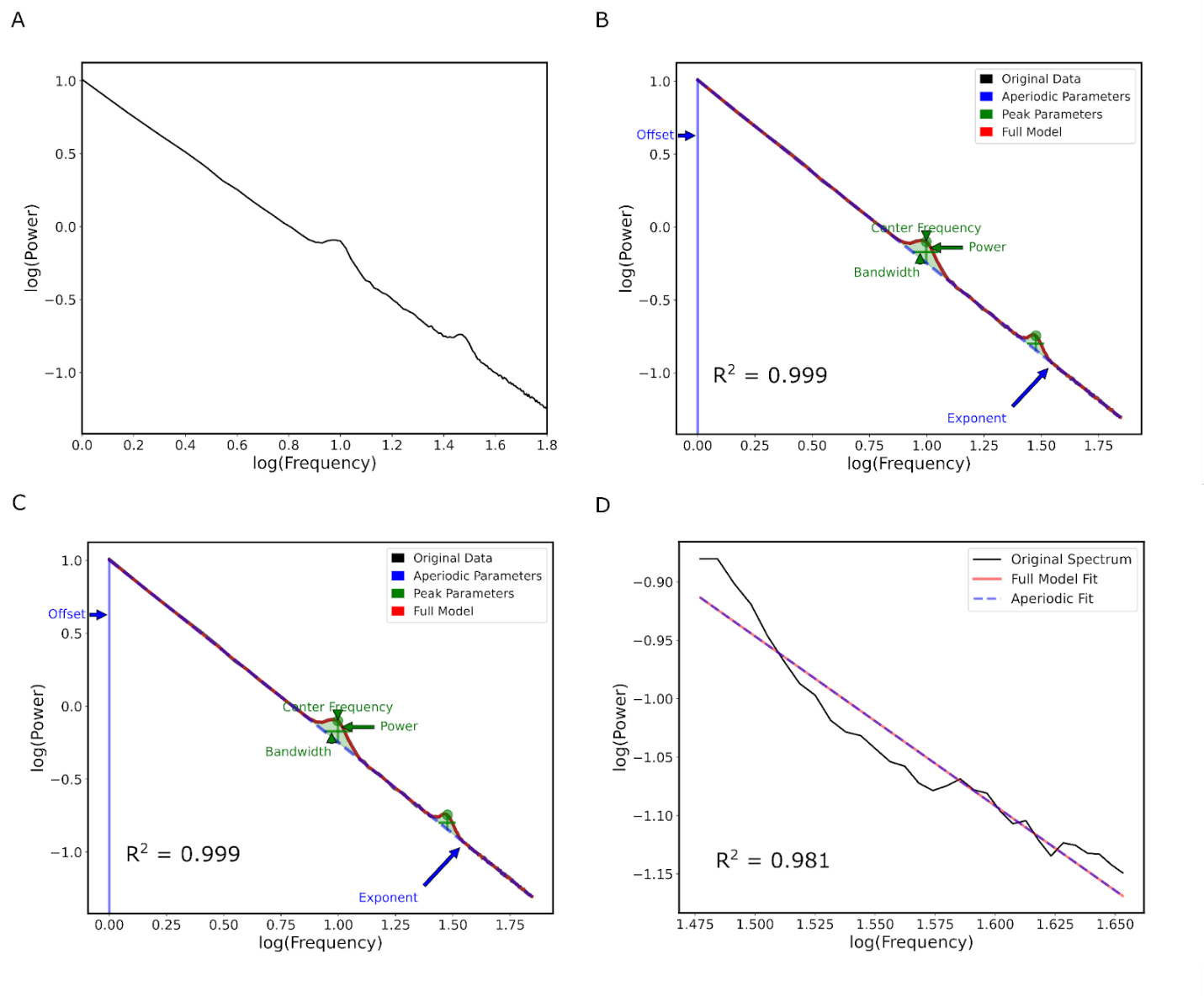
Supplementary Figure 2. Simulated signal with no knee.*** *A) Similar to Figure 1 we simulated a signal with two oscillatory peaks at 10 and 30 Hz. However, unlike Figure 1, we did not incorporate a knee. The knee model (B) and the fixed model with broad range (C) had comparable R^2^ and Exponent values. The fixed model with the narrow band (D) however, had lower R^2^ at 0.98. Note that even when no knee is present the knee model performed at high levels similar to the fixed model. R^2^: goodness-of-fit.*


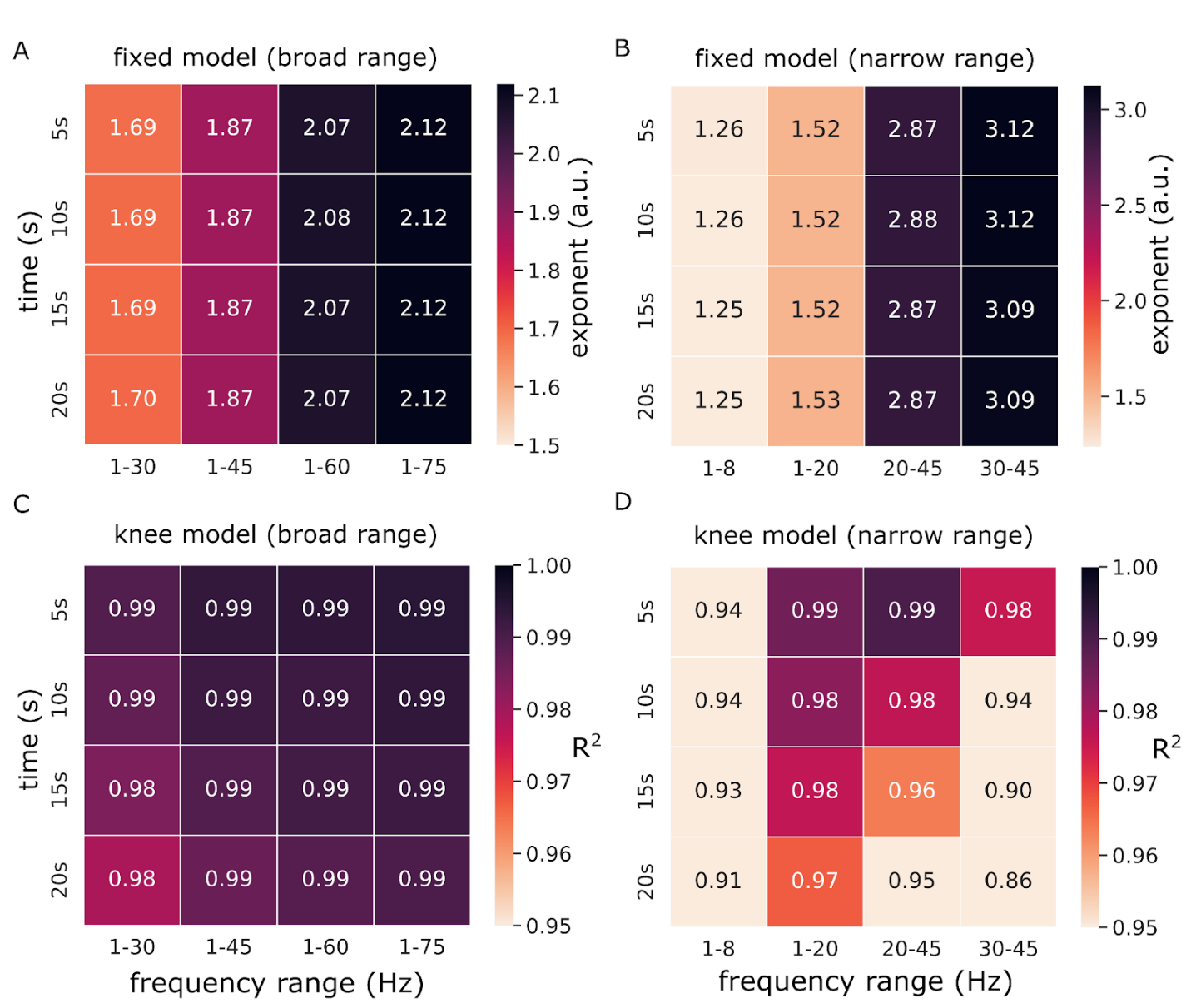
***Supplementary Figure 3. Model performance using broad and narrow frequency bands.*** *A-B) The exponent values resulting from using A) broad frequency ranges and B) narrow frequency bands. Note the higher variance in (B) as compared to (A).* *C-D) The goodness-of-fit (R^2^) of the knee model fitted to the iEEG data using C) broadband or D) narrow frequency bands. iEEG data were obtained from 38 brain regions across 106 subjects, with each region including a different subset of subjects.*

***
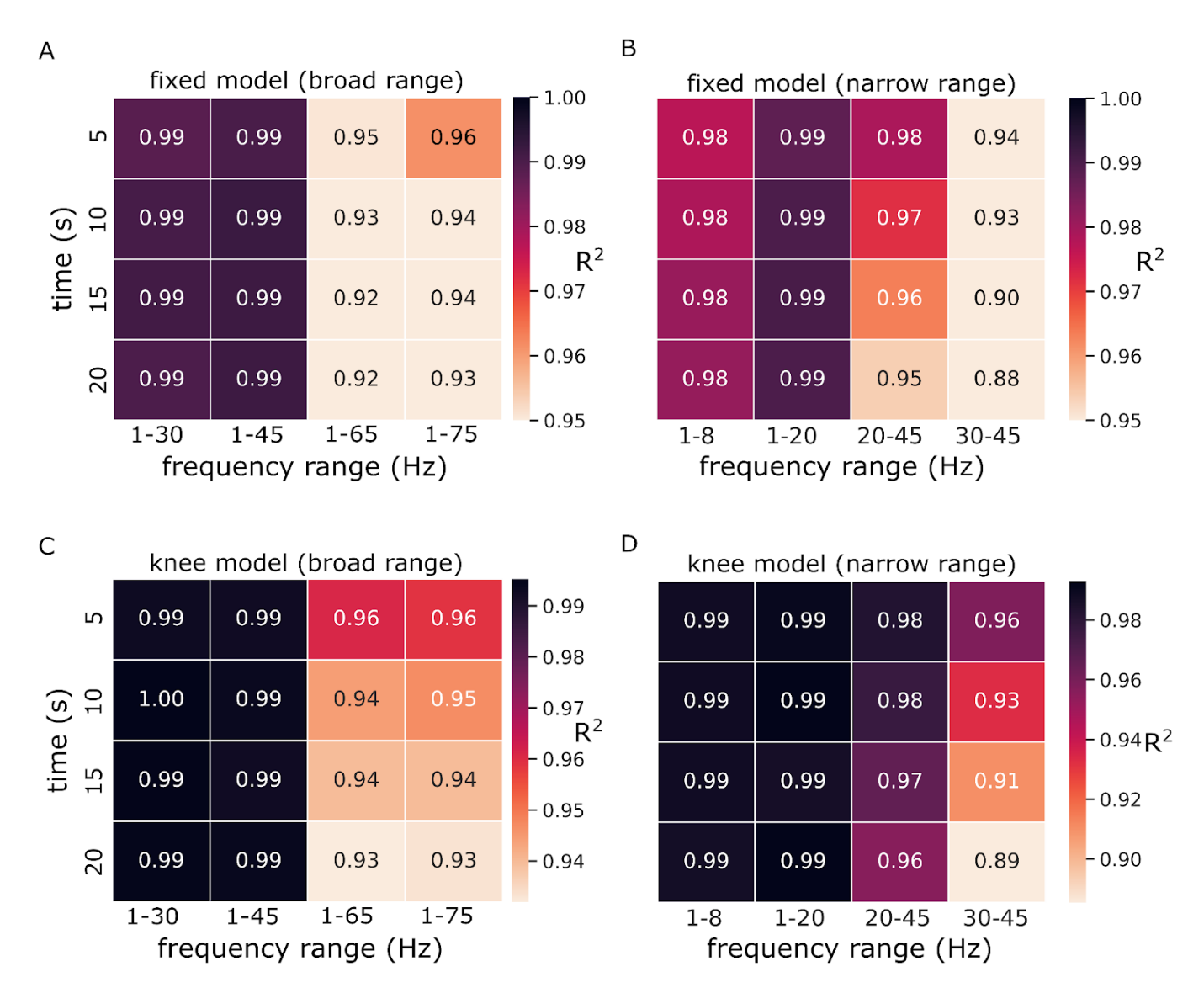
Supplementary Figure 4. Model performance for different frequency ranges in EEG.*** *A) The R^2^ values for the fixed model using broadband frequency ranges. B) The R^2^ values for the fixed model using narrow frequency ranges. C-D) The R^2^ values of the knee model using broadband (C) and narrowband (D) frequency ranges. iEEG data were obtained from 38 brain regions across 106 subjects, with each region including a different subset of subjects.*


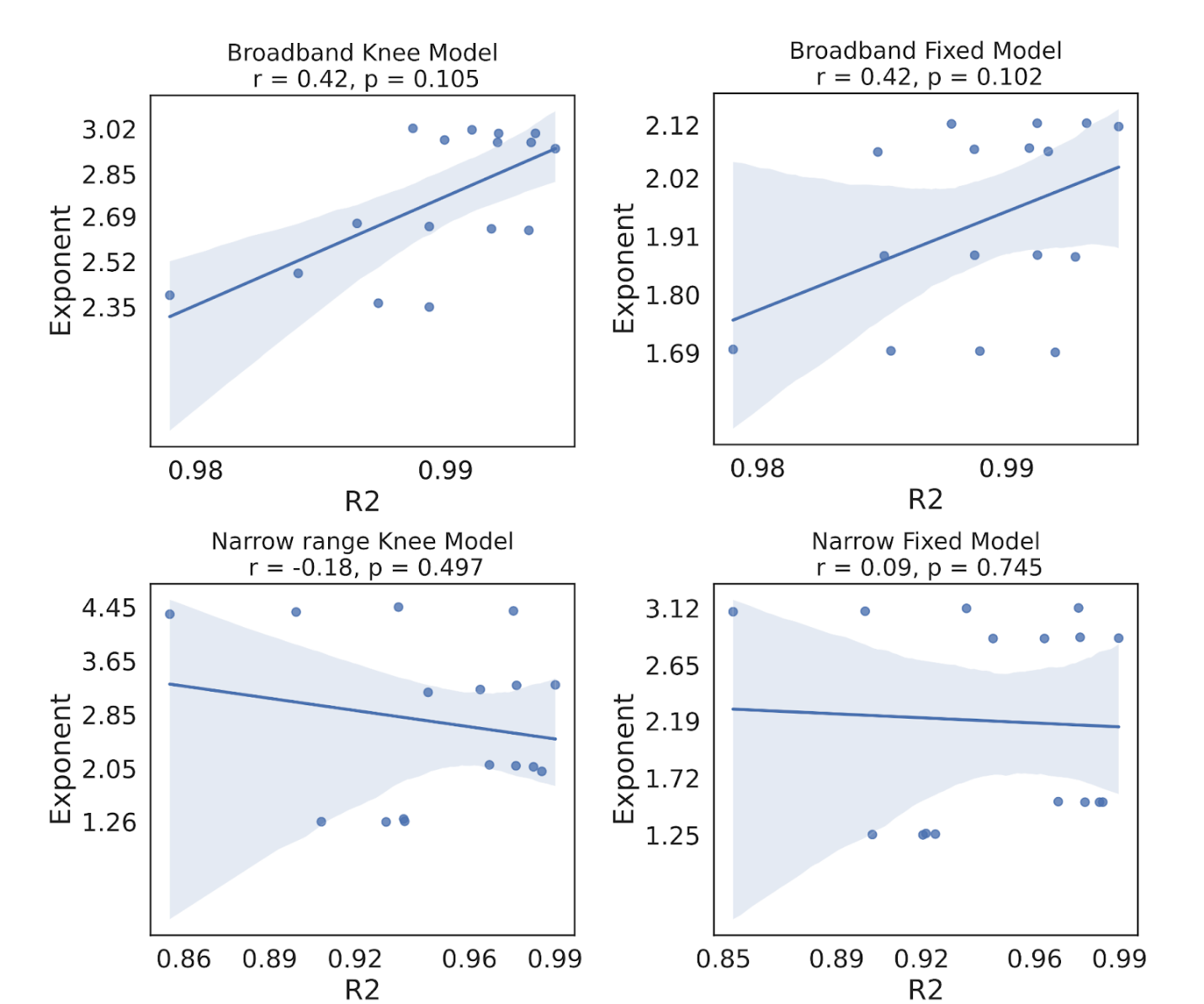
***Supplementary Figure 5. Correlation between spectral exponent and R² values across different models in the iEEG data.*** *Scatter plots illustrate the relationship between the spectral exponent and R² for four different model configurations: the Broadband Knee Model (top-left), the Broadband Fixed Model (top-right), the Narrow-range Knee Model (bottom-left), and the Narrow Fixed Model (bottom-right). Each plot includes a regression line with a 95% confidence interval. Spearman’s correlation coefficients (r) and p-values are displayed for each model. No significant correlation was found across any model, indicating the absence of a systematic bias or fitting artifact. iEEG data were obtained from 38 brain regions across 106 subjects, with each region including a different subset of subjects.*


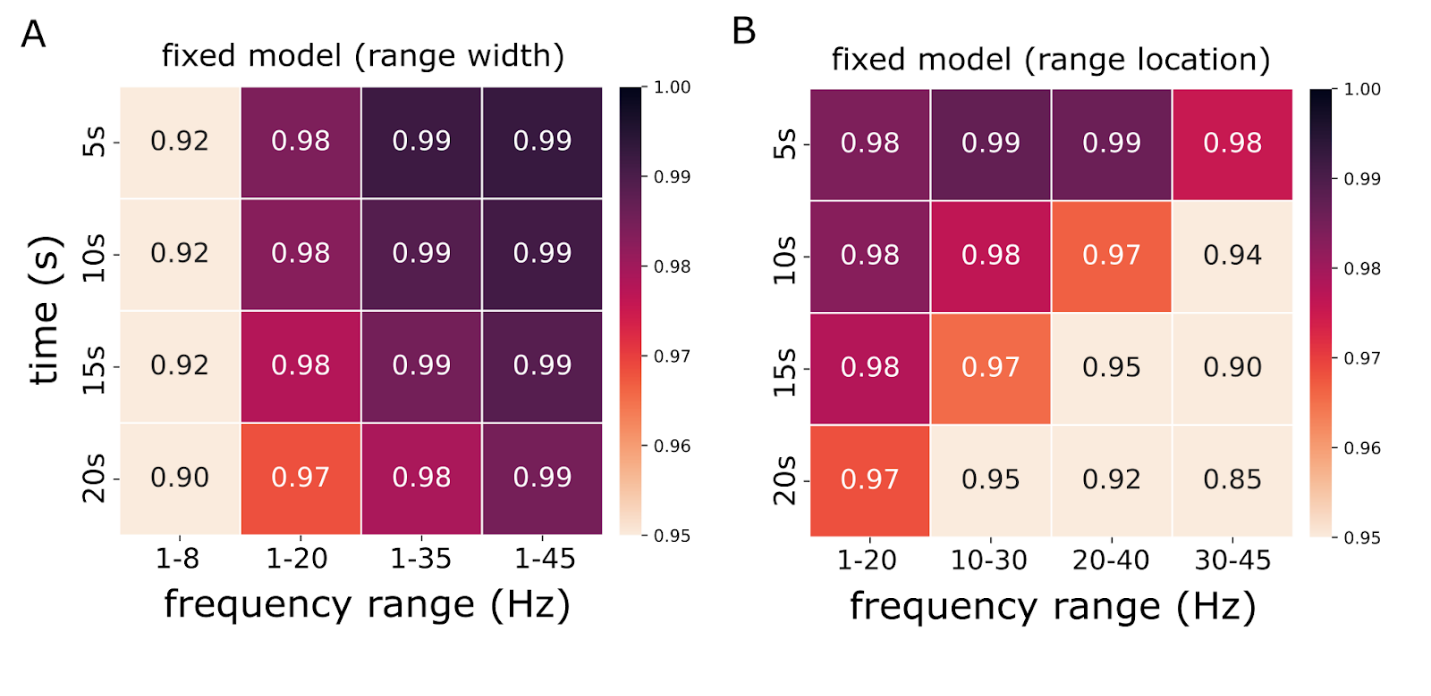
***Supplementary Figure 6. Effects of frequency range width and location on spectral model performance in iEEG data.*** *A-B) Sensitivity matrices showing R² values for models fitted across varying frequency ranges (x-axis) and time windows (y-axis). A) Increasing the width of the frequency band used for model fitting improved model performance, as indicated by higher R² values. B) Shifting the frequency band to higher frequency ranges reduced model fit quality, suggesting that excluding lower frequencies has a detrimental effect on model performance. iEEG data were obtained from 38 brain regions across 106 subjects, with each region including a different subset of subjects.*


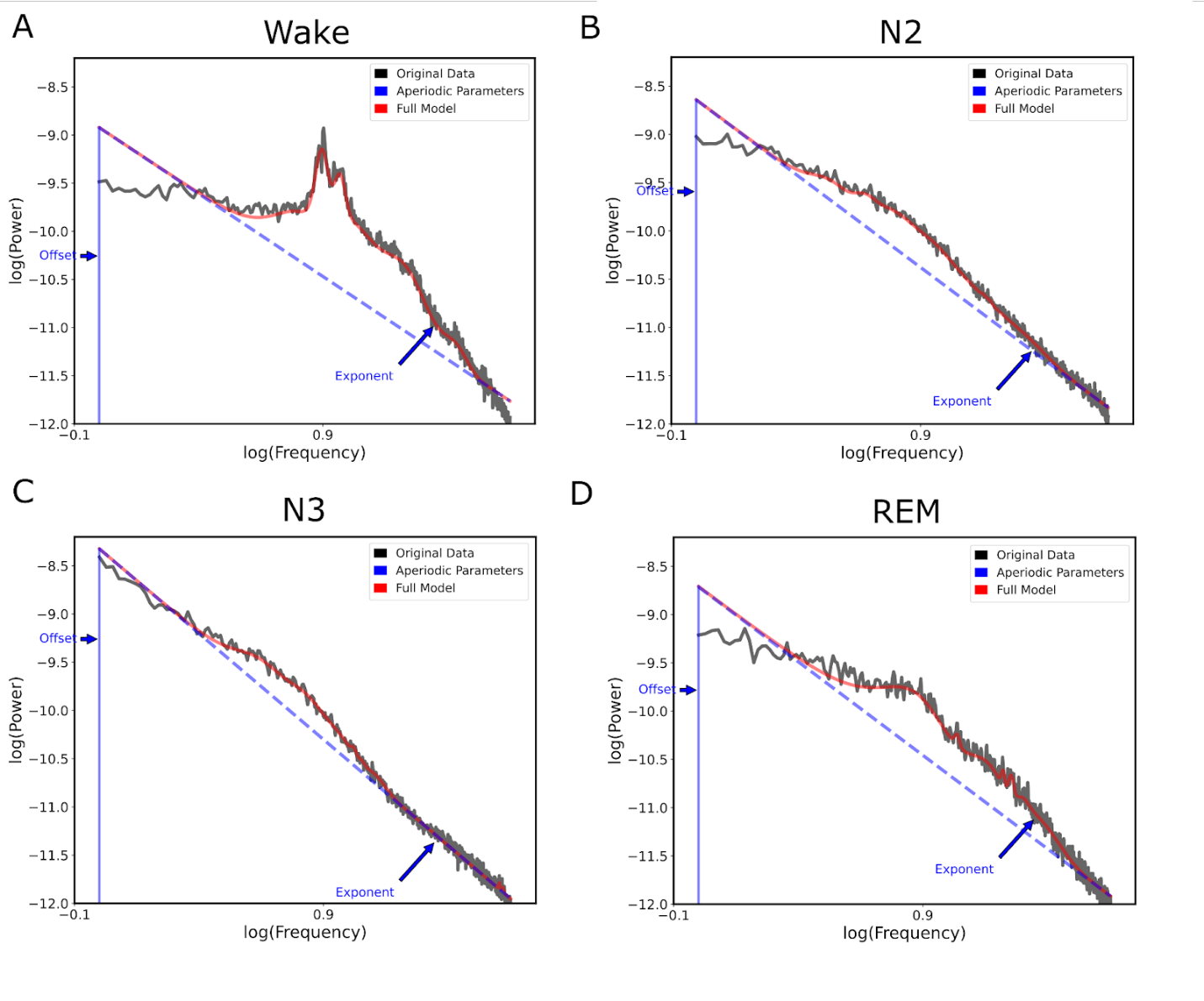
***Supplementary Figure 7. Model fit using a fixed model across a broad frequency range in the iEEG data.*** *The fixed model is fitted to A) Wake, B) N2, C) N3, and D) REM sleep data. iEEG data were obtained from 38 brain regions across 106 subjects, with each region including a different subset of subjects.*


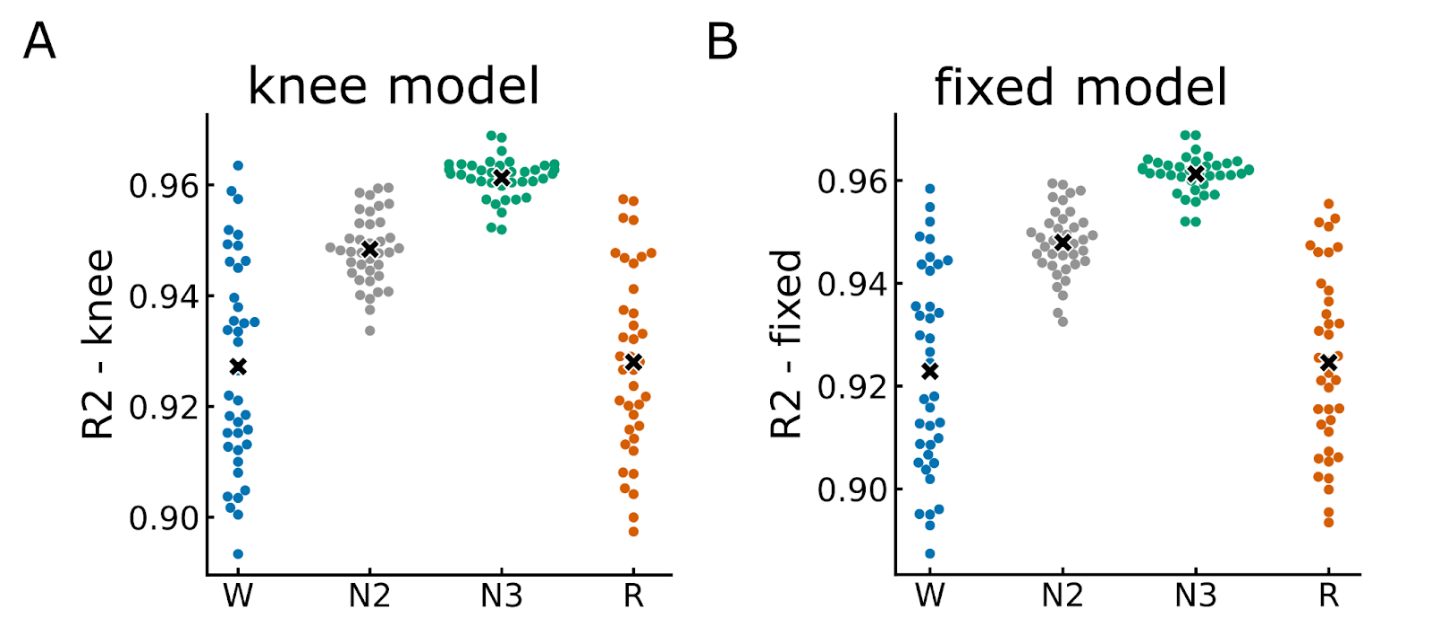
***Supplementary Figure 8. Model fit quality for the different sleep stages in the iEEG data.*** *R^2^ of model fit using either A) the knee model (broad range) or B) fixed model (broad range). Note the overall high R^2^ values for all models and all stages (R^2^ > 0.85). Also note the high variability for Wake and REM stages as compared to N2 and N3. iEEG data were obtained from 38 brain regions across 106 subjects, with each region including a different subset of subjects.*


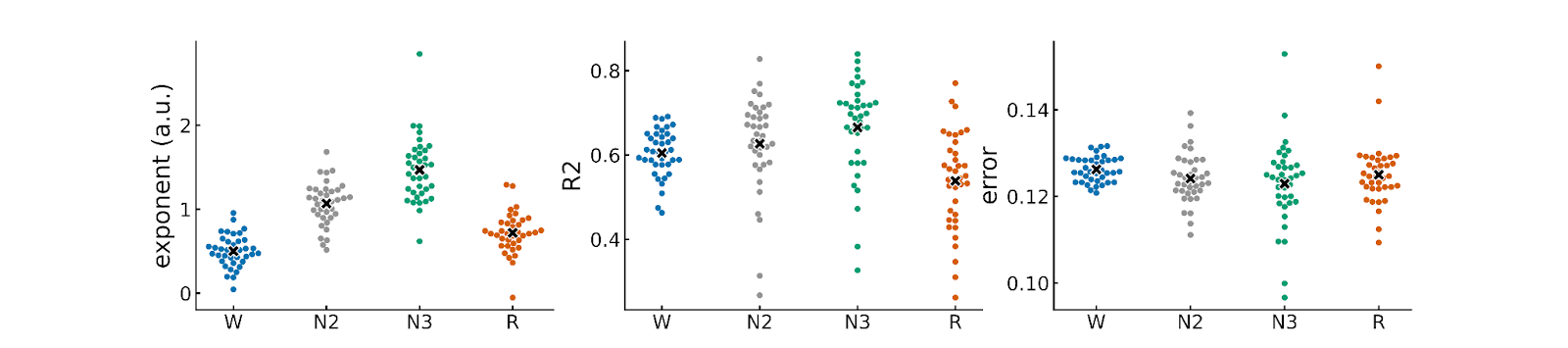


***Supplementary Figure 9. Sleep-stage differences in pre-knee exponent in iEEG data.*** *Trials where no knee was detected or knee frequency was below 2 Hz were excluded. The analysis is therefore done in 37 regions (excluding Amygdala as no trials met these conditions in at least one of the stages). The Friedman test revealed a significant difference between exponents of the different stages (*Friedman chi-square test: X^2^ = 96.02, p < 0.001, W = 0.87*). Post-hoc tests are reported in Table 5. iEEG data were obtained from 38 brain regions across 106 subjects, with each region including a different subset of subjects.*

*
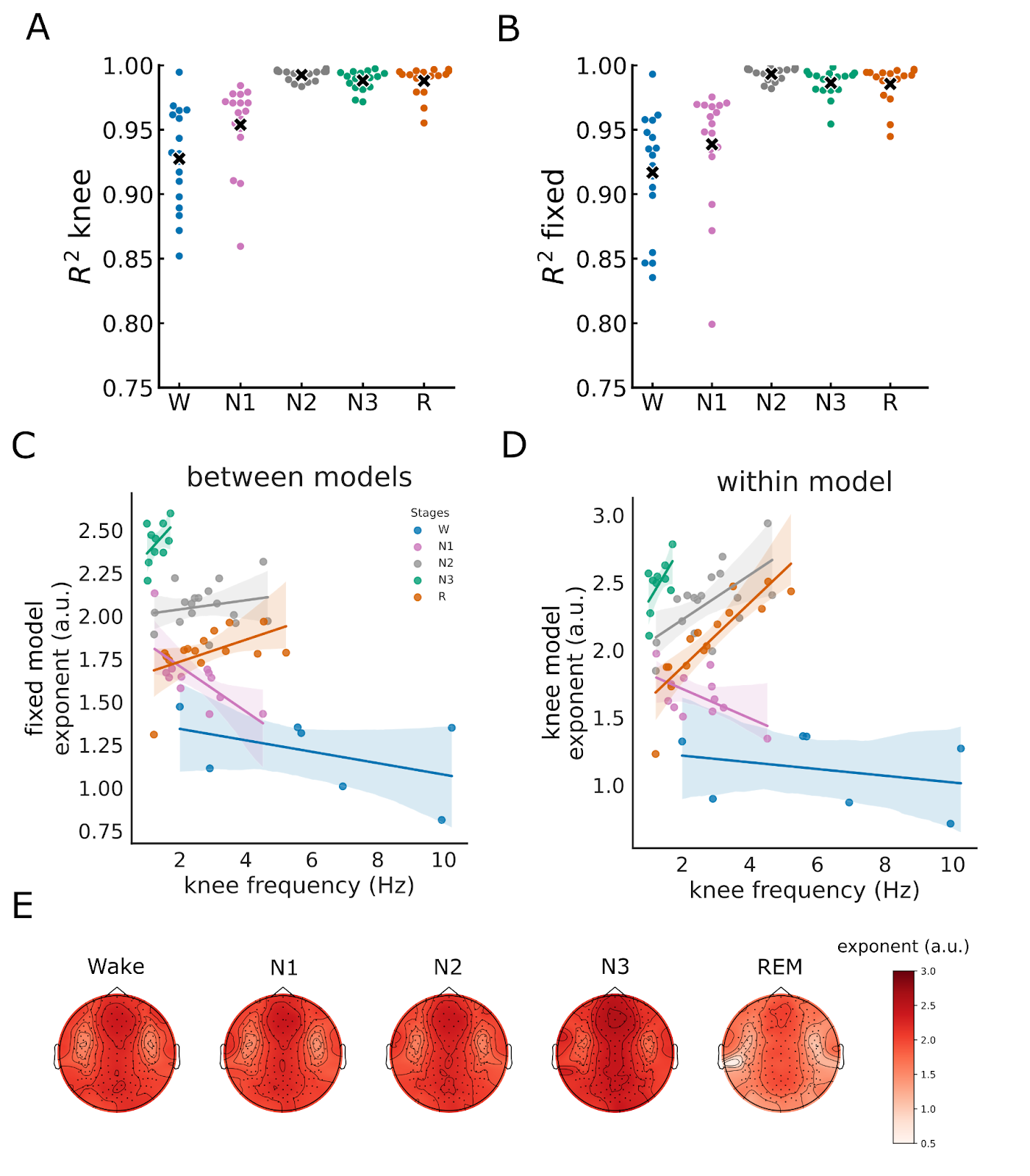
****Supplementary Figure 10. Extended EEG results.*** *A) R^2^ for the knee model for the different sleep stages. B) R^2^ of the fixed model for the different sleep stages. C-D) Partial correlations between knee frequency and exponents of knee and fixed models after correcting for sleep stages. C) Between-models correlation: The exponent of the fixed model correlated significantly negatively with the knee frequency derived from the knee model (Spearman correlation: rho(73) = -0.31, p = 0.006, 95% CI = [-0.5, -0.09]). D) Within-model correlation: The correlation between the knee frequency and the exponent of the knee model (Spearman correlation: rho(73) = -0.0007, p = 0.99, 95% CI = [-0.23, 0.23]). E) The topography of the exponent of the fixed model. Each dot in panels A, B, C, and D represents one subject. This analysis is based on EEG data collected from 17 healthy participants during overnight sleep.*


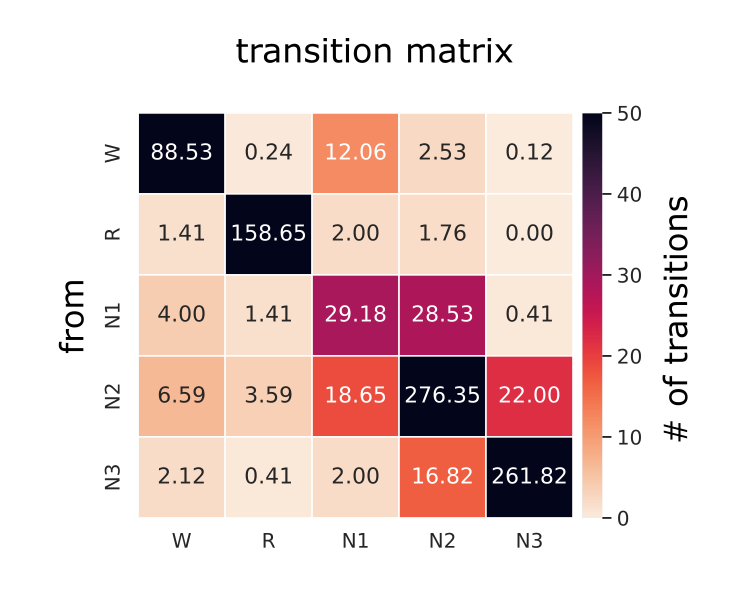
***Supplementary Figure 11. Matrix of transitions between sleep stages in EEG data****. Each cell represents the average number of transitions between two stages over all subjects. EEG data were recorded from 17 healthy participants during overnight sleep.*

***
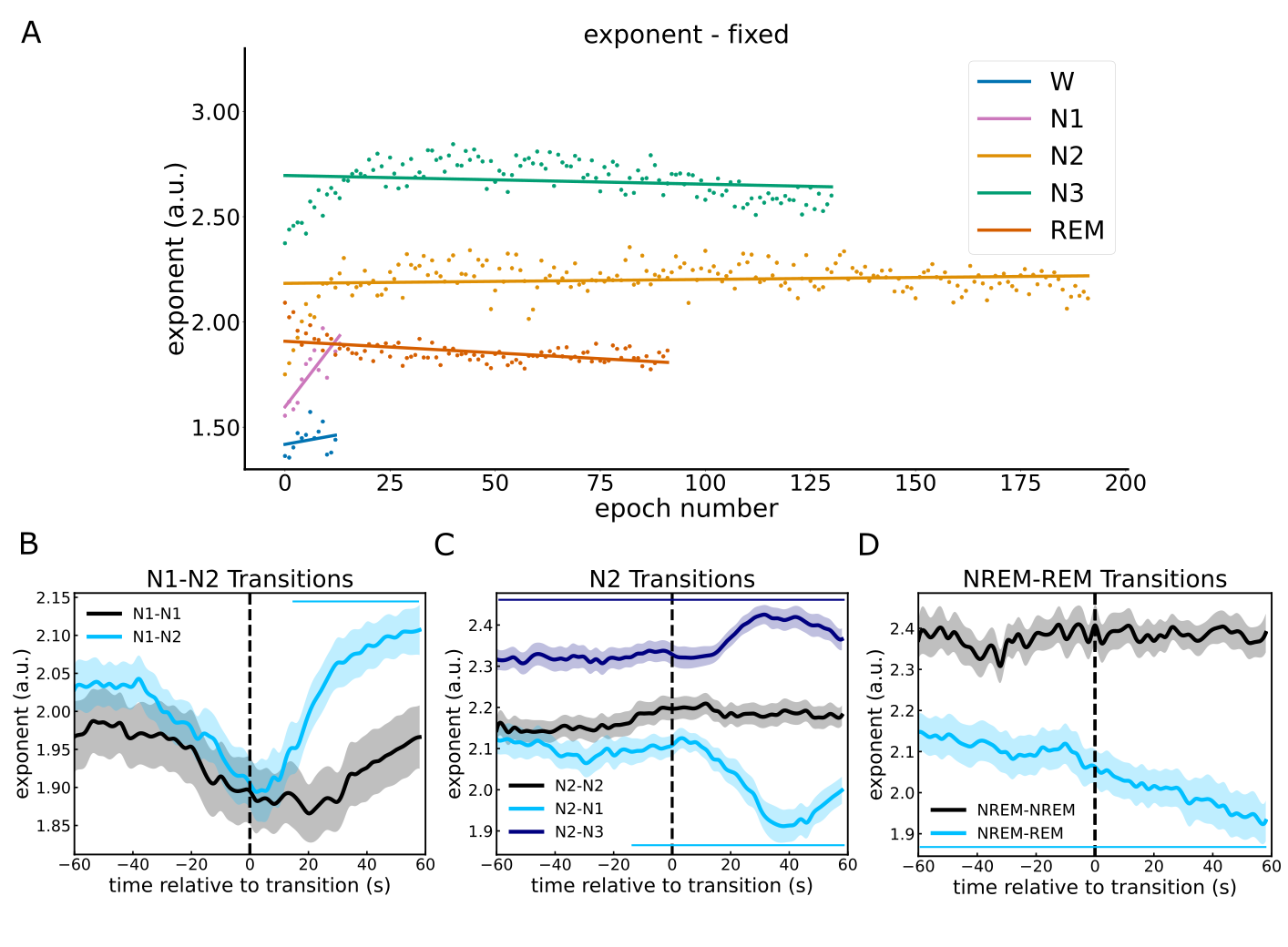
Supplementary Figure 12. Replication of Figure 6 using the exponent of the fixed model. A)*** *Epoch-by-epoch analysis of the exponent value throughout the night for each sleep stage. The results revealed for N1 and REM sleep that the exponent changed significantly with the passage of time. The N1 exponent increased significantly N1: R^2^ = 0.7, F(1,12) = 27.59, p < 0.001) while that of REM decreased significantly (REM: R^2^ = 0.3, F(1,90) = 37.9, p = 0.01). For Wake, N2 and N3, however, the change in the exponent over time did not reach statistical significance (Wake: R^2^ = 0.05, F(1,11) = 0.54, p = 0.48 - N2: R^2^ = 0.01, F(1,190) = 0.54, p = 0.09 - N3: R^2^ = 0.03, F(1,29) = 3.58, p = 0.06). B-D) Exponent change around the transitions between sleep stages. B) Comparing the transitions from N1 to N2 against a baseline of continuous N1 showed that the exponent increased after the transition from N1 to N2 reaching a significant difference to that of the baseline starting at 14 s following the transition (14s - 60s: ∑t(17) = 147.18, p < 0.001, d = 1.7). C) Examining the transitions from N2 to either N3 or N1. The results revealed a significant difference in the exponent between N2-N1 and N2-N2 that started 12 s before the transition (-12s - 60s: ∑t(16) = 237.5, p < 0.001, d = -2.33). The exponent during N2 to N3 was significantly different from the baseline during the whole duration of the segment (-60s - 60s: ∑t(16) = 515.28, p < 0.001, d = 2.56), making it difficult to map the temporal dynamics of this transition. D) The exponent during the transitions from NREM to REM sleep showed a significant deviation from the baseline throughout the entire segment, with a substantial statistical difference ( -60s - 60s: ∑t(16) = 335.16, p < 0.001, d = 1.69). These marked variances in C and D presented a challenge in accurately mapping the temporal dynamics associated with this sleep stage transition. The EEG data were recorded from 17 healthy participants during overnight sleep.*


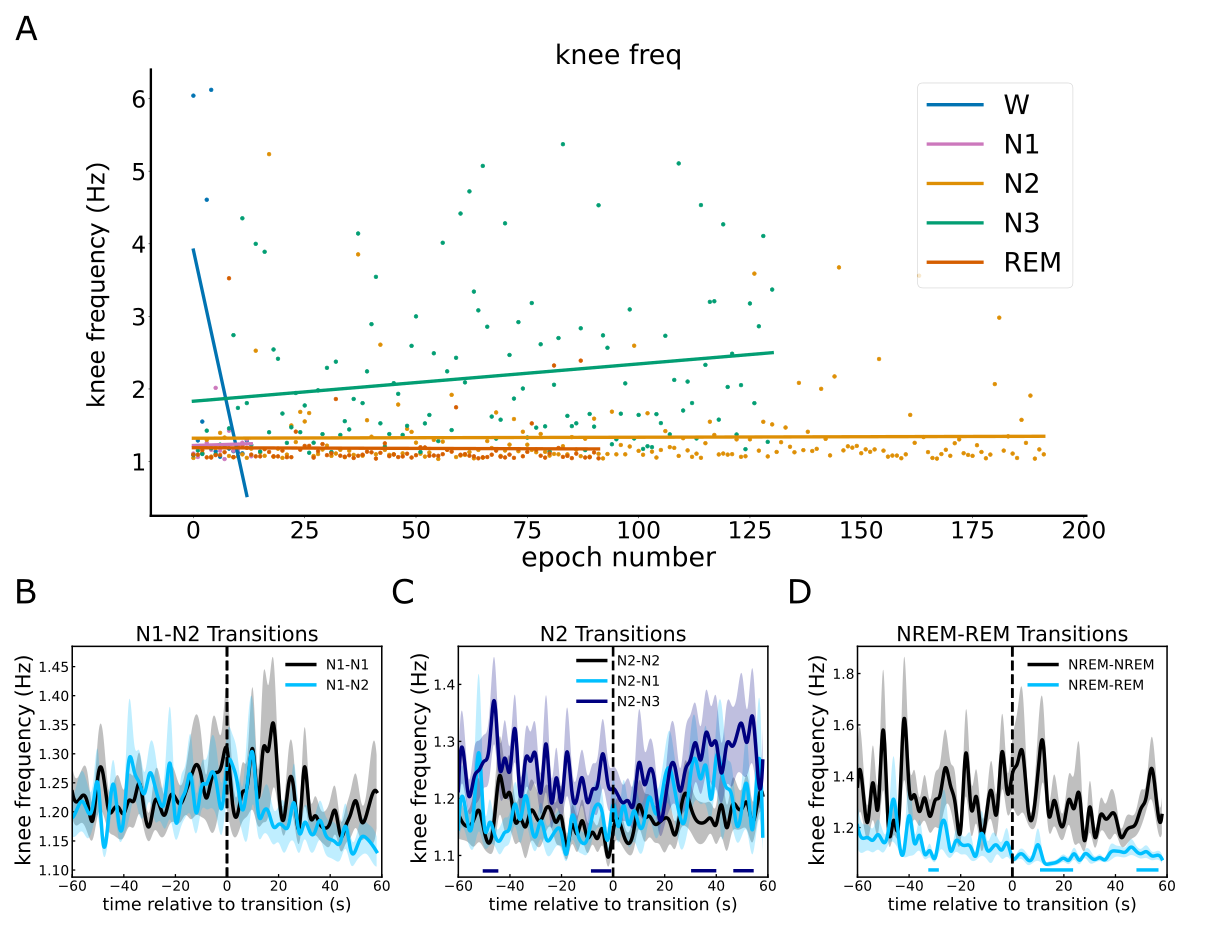
***Supplementary Figure 13. Replication of Figure 6 using the knee frequency.*** *A)* *Epoch-by-epoch analysis of the exponent value throughout the night revealed a significant decrease in the knee frequency across wake epochs (R^2^ = 0.32, F(1,11) = 25.06, p = 0.046) and a significant increase across N3 epochs (R^2^ = 0.04, F(1,129) = 4.71, p = 0.03). N1, N2 and REM, however, showed no significant change in the knee frequency with the passage of time (N1: R^2^ = 0.001, F(1,12) = 0.01 p = 0.91, N2: R^2^ = 0.0002, F(1,190) = 27.59, p = 0.84, REM: R^2^ = 0.0002, F(1,90) = 0.02, p = 0.89). B-D) Temporally resolved estimates of the knee frequency during transitions between sleep stages. B) No difference observed in the knee frequency during the transition from N1 to N2 as compared to a continuous N1 baseline (p > 0.1). C) The transitions from N2 to either N3 or N1. No difference between the transitions from N2 to N1 and the baseline (continuous N2, p > 0.05). For the transition from N2 to N3 however, four significant clusters were observed (-50s - -46s: ∑t(16) = 9.54, p = 0.01, d = 0.94, -6s - 0s: ∑t(16) = 9.98, p = 0.009, d = 0.78, 32s - 40s: ∑t(16) = 13.08, p = 0.004, d = 0.8, 48s - 54s: ∑t(16) = 11.42, p = 0.006, d = 0.88). D) NREM to REM transitions as compared to a NREM baseline differed significantly at three periods (-34s - 30s: ∑t(16) = 13.08, p = 0.004, d = 0.79, 12s - 22s: ∑t(16) = 15.18, p = 0.001, d = 0.88, 48s - 56s: ∑t(16) = 13.23, p = 0.004, d = 0.76). EEG data were recorded from 17 healthy participants during overnight sleep.*


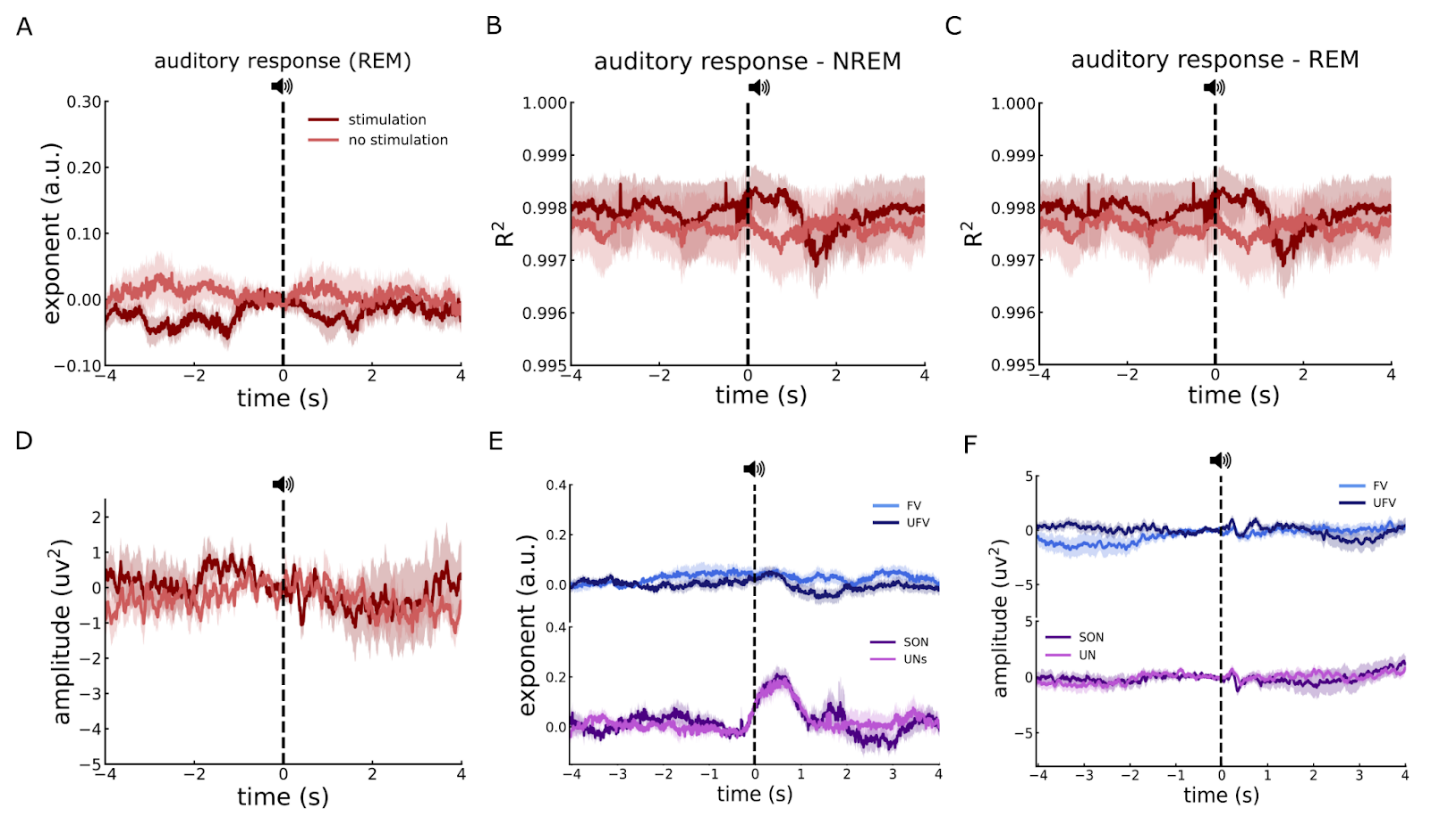


***Supplementary Figure 14****.* ***Auditory responses during REM sleep.*** *A) Time-resolved estimates of aperiodic exponent in response to auditory stimuli, during REM sleep. B-C) The goodness-of-fits (R^2^) for fits around the presentation of auditory stimuli during (B) NREM and (C) REM. D-F) Auditory responses to different stimuli during REM sleep. D) Auditory ERPs during REM did not differ from a baseline of no stimulation. E-F) During REM sleep, when examining the responses to various voices and names, we found no significant differences in the exponent values for (E-top) different voices and (E-bottom) different names. Similarly, the ERPs also did not exhibit any notable variation between the different (F-top) voices and (F-bottom) names. Dashed vertical lines at time zero represent stimulus onset. This analysis was performed on EEG data that were recorded from 17 healthy participants during overnight sleep.*


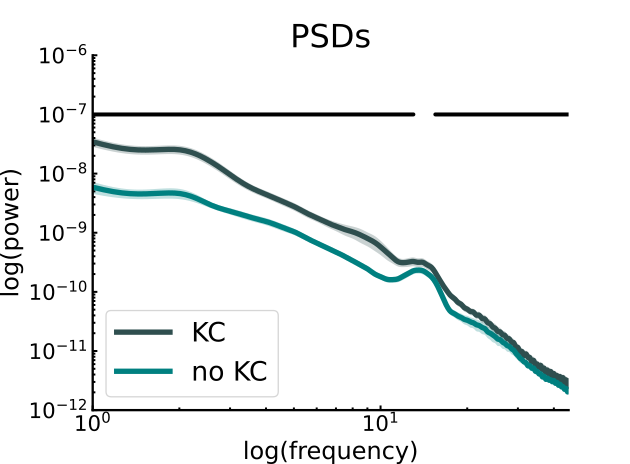


***Supplementary Figure 15. PSDs of the KC vs no KC analysis.*** *We identified the peak points of exponent responses to auditory stimuli under two conditions: when a KC was evoked and when it was not. At these peak moments, we calculated the PSDs. It is important to note the observed broadband variation in power, which was higher in trials where a KC was evoked. This suggests a shift in the aperiodic exponent rather than a difference that is attributed to oscillatory changes. The horizontal lines indicate the significant clusters. This analysis was performed on EEG data that were recorded from 17 healthy participants during overnight sleep.*

69. Alnes, S. L., Bächlin, L. Z. M., Schindler, K., & Tzovara, A. (2023). Neural complexity and the spectral slope characterise auditory processing in wakefulness and sleep. The European Journal of Neuroscience.<https://doi.org/10.1111/ejn.16203>

70. Ameen, M. S., Petzka, M., Peigneux, P., & Hoedlmoser, K. (2023). Post-training sleep modulates motor adaptation and task-related beta oscillations. Journal of Sleep Research, e14082.<https://doi.org/10.1111/jsr.14082>

71. Andrillon, T., Solelhac, G., Bouchequet, P., Romano, F., Le Brun, M.-P., Brigham, M., Chennaoui, M., & Léger, D. (2020). Revisiting the value of polysomnographic data in insomnia: More than meets the eye. *Sleep Medicine*, *66*, 184–200.<https://doi.org/10.1016/j.sleep.2019.12.002>

72. Favaro, J., Colombo, M. A., Mikulan, E., Sartori, S., Nosadini, M., Pelizza, M. F., Rosanova, M., Sarasso, S., Massimini, M., & Toldo, I. (2023). The maturation of aperiodic EEG activity across development reveals a progressive differentiation of wakefulness from sleep. NeuroImage, 277, 120264.<https://doi.org/10.1016/j.neuroimage.2023.120264>

73. Maschke, C., Duclos, C., Owen, A. M., Jerbi, K., & Blain-Moraes, S. (2023). Aperiodic brain activity and response to anesthesia vary in disorders of consciousness. NeuroImage, 275, 120154.<https://doi.org/10.1016/j.neuroimage.2023.120154>

74. Wen, H., & Liu, Z. (2016). Separating Fractal and Oscillatory Components in the Power Spectrum of Neurophysiological Signal. Brain Topography, 29(1), 13–26.<https://doi.org/10.1007/s10548-015-0448-0>
