# Supplementary material for: Temporally resolved analyses of aperiodic features track neural dynamics during sleep
Source: Commun Psychol. 2025 Nov 19;3:160. doi: 10.1038/s44271-025-00334-2 (PMC12630853; doi:10.1038/s44271-025-00334-2)
Supplement: Supplementary file 3 — Reporting Summary [file 44271_2025_334_MOESM3_ESM.pdf]

nature portfolio

Corresponding author(s): Mohamed S. Ameen

Last updated by author(s): Sep 25, 2025

Reporting Summary

Nature Portfolio wishes to improve the reproducibility of the work that we publish. This form provides structure for consistency and transparency in reporting. For further information on Nature Portfolio policies, see our Editorial Policies and the Editorial Policy Checklist.

Statistics

For all statistical analyses, confirm that the following items are present in the figure legend, table legend, main text, or Methods section.

n/a	Confirmed	
☐	☒	The exact sample size (*n*) for each experimental group/condition, given as a discrete number and unit of measurement
☐	☒	A statement on whether measurements were taken from distinct samples or whether the same sample was measured repeatedly
☐	☒	The statistical test(s) used AND whether they are one- or two-sided<br>*Only common tests should be described solely by name; describe more complex techniques in the Methods section.*
☐	☒	A description of all covariates tested
☐	☒	A description of any assumptions or corrections, such as tests of normality and adjustment for multiple comparisons
☐	☒	A full description of the statistical parameters including central tendency (e.g. means) or other basic estimates (e.g. regression coefficient) AND variation (e.g. standard deviation) or associated estimates of uncertainty (e.g. confidence intervals)
☐	☒	For null hypothesis testing, the test statistic (e.g. F, t, r) with confidence intervals, effect sizes, degrees of freedom and P value noted<br>*Give P values as exact values whenever suitable.*
☒	☐	For Bayesian analysis, information on the choice of priors and Markov chain Monte Carlo settings
☐	☒	For hierarchical and complex designs, identification of the appropriate level for tests and full reporting of outcomes
☐	☒	Estimates of effect sizes (e.g. Cohen's d, Pearson's r), indicating how they were calculated

Our web collection on statistics for biologists contains articles on many of the points above.

Software and code

Policy information about availability of computer code

Data collection	Open source iEEG data from the MNI Atlas: https://mni-open-ieegatlas.research.mcgill.ca/
Data analysis	Specparam (Formerly FOOOF) toolbox was used (Donoghue et al., 2020) and cited whenever appropriate throughout the manuscript

For manuscripts utilizing custom algorithms or software that are central to the research but not yet described in published literature, software must be made available to editors and reviewers. We strongly encourage code deposition in a community repository (e.g. GitHub). See the Nature Portfolio guidelines for submitting code & software for further information.

Data

Policy information about availability of data

All manuscripts must include a data availability statement. This statement should provide the following information, where applicable:

- Accession codes, unique identifiers, or web links for publicly available datasets
- A description of any restrictions on data availability
- For clinical datasets or third party data, please ensure that the statement adheres to our policy

Data is available upon request

Human research participants

Policy information about studies involving human research participants and Sex and Gender in Research.

Reporting on sex and gender	We recruited 20 healthy participants with no reported history of neurological or psychological problems as well as no reported sleep disorders. However, one participant dropped out after the adaptation night, and we had to exclude two participants because of technical problems during EEG acquisition. Therefore,we performed the analyses on 17 participants (14 females) with a median age of 22.6 +/- 2.3 years. Before beginning the experiment, all participants signed written informed consents. The experiment was approved by the Ethics Committee of the University of Salzburg.
Population characteristics	see above
Recruitment	For the EEG part, participants were recruited from the student population of the University of Salzburg and through local advertisement
Ethics oversight	Ethics department of the university of Salzburg

Note that full information on the approval of the study protocol must also be provided in the manuscript.

Field-specific reporting

Please select the one below that is the best fit for your research. If you are not sure, read the appropriate sections before making your selection.

☒ Life sciences ☐ Behavioural & social sciences ☐ Ecological, evolutionary & environmental sciences

For a reference copy of the document with all sections, see nature.com/documents/nr-reporting-summary-flat.pdf

Life sciences study design

All studies must disclose on these points even when the disclosure is negative.

Sample size	Sample size was determined based on the time planned for recruiting participants. As this was an exhaustive protocol were participants have to sleep in twice a sleep lab for a full night with EEG, we ended up recruiting 20 participants.
Data exclusions	Two participants were excluded (19-2 = 17) due to technical or personal issues. The exclusion of outliers in the analysis is reported in the methods part of the main manuscript and was based on the standard deviation calculated over the whole sample.
Replication	All codes were uploaded to GitHub. All steps of statistical analyses were reported thoroughly and effect sizes for all analyses were reported. Additionally, raw data would be available upon request.
Randomization	There is no randomization in our study as we are using a within-subject study design
Blinding	There is no group allocation in our study

Reporting for specific materials, systems and methods

We require information from authors about some types of materials, experimental systems and methods used in many studies. Here, indicate whether each material, system or method listed is relevant to your study. If you are not sure if a list item applies to your research, read the appropriate section before selecting a response.

Materials & experimental systems

n/a	Involved in the study	
☒	☐	Antibodies
☒	☐	Eukaryotic cell lines
☒	☐	Palaeontology and archaeology
☒	☐	Animals and other organisms
☒	☐	Clinical data
☒	☐	Dual use research of concern

Methods

n/a	Involved in the study	
☒	☐	ChIP-seq
☒	☐	Flow cytometry
☒	☐	MRI-based neuroimaging
